# Immersive Virtual Reality for Health Promotion and Primary Prevention in Psychology: Scoping Review

**DOI:** 10.2196/49923

**Published:** 2025-01-20

**Authors:** Solenne Bonneterre, Oulmann Zerhouni, Marilisa Boffo

**Affiliations:** 1 Laboratoire Parisien de Psychologie Sociale, Département de Psychologie, Université Paris Nanterre, Nanterre, France; 2 Centre de Recherche sur les Fonctionnements et Dysfonctionnements Psychologiques, UR 7475, Université de Rouen Normandie, Rouen, France; 3 Department of Psychology, Education, and Child Studies, Erasmus School of Social and Behavioral Sciences, Erasmus University Rotterdam, Rotterdam, Netherlands

**Keywords:** virtual reality, health psychology, prevention psychology, health promotion

## Abstract

**Background:**

Virtual reality (VR) has emerged as a promising tool in health promotion and prevention psychology. Its ability to create immersive, engaging, and standardized environments offers unique opportunities for interventions and assessments. However, the scope of VR applications in this field remains unclear.

**Objective:**

This scoping review aims to identify and map the applications of VR in health promotion and prevention psychology, focusing on its uses, outcomes, and challenges.

**Methods:**

A systematic search was conducted across 3 electronic databases (PubMed, PsycINFO, and Scopus) for studies published between 2010 and 2024. Eligibility criteria included empirical studies using immersive VR for health promotion and prevention, while studies using nonimmersive VR, lacking health-related applications, or focusing on clinical interventions were excluded. The review followed PRISMA-ScR (Preferred Reporting Items for Systematic Reviews and Meta-analyses extension for Scoping Reviews) guidelines, and 4295 records were initially identified, with 51 studies included after screening. Data were synthesized qualitatively to identify key applications, limitations, and emerging trends.

**Results:**

VR was primarily used in three areas: (1) delivering interventions (eg, pilot testing, skills training), (2) exploring fundamental research questions, and (3) assessing outcomes such as behavioral or psychological responses. Although VR demonstrated potential for enhancing user engagement and replicating ecological scenarios, its effectiveness compared to nonimmersive methods varied. Most studies were pilot or feasibility studies with small, nonrepresentative samples, short follow-up periods, and limited methodological standardization.

**Conclusions:**

VR offers a versatile and promising tool for health promotion and prevention but its applications are still in the early stages. The evidence is limited by methodological weaknesses and variability in outcomes. Future research should prioritize replication, longitudinal designs, and standardized methodologies to strengthen the evidence base and expand the applicability of VR interventions.

## Introduction

### Background

Health and prevention psychology aims to address health-related issues to either prevent individuals from starting or continuing an unhealthy behavior (ie, primary prevention), help them to detect or reduce illness in early stages (ie, secondary prevention), or support individuals in their journey against consequences of heavier injuries or diseases (ie, tertiary prevention, [1]). Although secondary and tertiary prevention are more individual-based depending on the illness or signs or symptoms individuals need to learn to cope with, primary prevention is broader and aimed at a larger audience. Therefore, primary or universal prevention is designed to prevent individuals from the general population from getting injured or sick and aims to enable people to live a sustainable and healthy lifestyle [2,3].

In this sense, health promotion campaigns have started to integrate technological innovations such as virtual reality (VR). We refer to VR as a type of human-computer interface immersing users into a computer-generated 3D virtual environment (VE) they can interact with in a naturalistic fashion, usually via an avatar (ie, representation of the user in the VE [4]). More pragmatically, we labeled as VR any type of device that has the ability to sensorily detach the user from the outside world (at least sight, but also sounds, smell, and touch in some cases). This includes the use of a cave automatic VE (users are surrounded by walls displaying the VE) or a head-mounted display (HMD), which blocks the user’s field of view outside of the VE and from which the user cannot turn away by simply looking away (ie, computer screens or 360° videos will not be considered VR in this definition).

The main aim of VR is to recreate a realistic, ecological context and experience while keeping some degree of experimental control over it [5-7]. Systematic reviews have reported promising results from VR-based interventions in other disciplines (eg, clinical psychology [8] and social psychology [9,10]). However, to our knowledge, there has been no review of the use of VR technologies for primary health promotion and prevention. Therefore, instead of focusing on specific research questions related to a topic, outcome, or population, the goal of this review was to map the current state of the art of the use of VR in such areas and identify gaps and future directions.

### Rationale

#### Virtual Reality: Operating Principles

The VR literature highlights 2 essential concepts, immersion and presence, both of which are critical to the user’s experience in VEs [7,11]. Immersion refers to the technological ability of a VR system to fully engage the user by replacing real-world sensory inputs with virtual stimuli. The more immersive the device, the less interface there is between the user and the virtual world. High immersion includes naturalistic interactions, such as the use of body suits to track movement, which increases the sense of realism [7]. Immersive systems create a sense that the virtual world is an actual experience rather than a mediated one. However, presence depends on the user’s psychological response to the VE. It is the subjective feeling of “being there” in the virtual world, interacting with it as if it were real [12]. This sense of presence increases engagement and leads to more vivid, memorable experiences [13]. Notably, presence can be felt in both immersive and nonimmersive media, such as movies or books, as it is influenced by individual factors and not just the technological features of the medium [14].

Although immersion and presence are often related, they are not the same. Higher levels of immersion tend to enhance feelings of presence, but immersion is not a necessary condition for presence [15]. Thus, immersion can be viewed as a moderator that enhances presence but does not guarantee it [16].

### Why Use VR in Health Promotion and Prevention Psychology?

VR technology has emerged as a promising tool in health promotion and prevention psychology, allowing for immersive experiences that can enhance user engagement and motivation [17,18]. VR enables researchers to create safe, ecological, and standardized VEs, where health promotion interventions can be effectively delivered and evaluated. VR presents key advantages as a tool for research and intervention in health promotion and primary prevention [7].

First, VR can be combined with devices aimed at mimicking more natural movements (eg, the use of handheld controllers or haptic devices instead of a mouse and keyboard) and can encompass the integration of full-body motor and haptic feedback when using a bodysuit. This freedom and wholeness of movement can help enhance learning through direct practice, visualization, and ultimately embodied cognition (ie, cognition linked to the body [7,19]). Hence, VR can be a relevant tool to create interventions aimed at learning health-related behaviors that require practicing skills (eg, detecting testicular disorders [20]).

Second, due to its ability to elicit embodiment, VR is well suited to elicit and enhance perspective-taking and empathy [7,21]. For example, embodying an obese avatar could enhance taking the perspective of being overweight, leading to a more effective learning of the consequences of obesity and, in turn, a greater intention to take care of individual health (ie, reduce the attitude-intention-behavior gap). Through the feeling of presence, individuals can visualize themselves in a specific situation, hence allowing a deeper sense of self-reflection [20], potentially leading to more persistent changes in behavior. VR can recreate ecological situations and environments in which users can embody an avatar and act in the virtual world as if it were real, through the feeling of presence [12].

### Objective

Our goal was to identify and map how VR has been used in the field of health promotion and primary prevention. In this scoping review, we addressed three broad research questions:

1. What are the uses of VR technology in primary prevention and health promotion (ie, an overview of the goals and research questions addressed through the use of VR)?
2. What do we know so far about the effects of using VR in these fields (ie, a summary of the results)?
3. What are the challenges and limitations, if any, encountered so far?

Based on the findings of the scoping review, we drafted a list of recommendations and perspectives for the use of VR in health promotion and primary prevention.

## Methods

### Protocol and Registration

The scoping review protocol was drafted according to the PRISMA-ScR (Preferred Reporting Items for Systematic Reviews and Meta-analyses extension for Scoping Reviews) checklist [22,23]. We also conducted a synthesis without meta-analysis [24] (Checklist 1).

### Eligibility Criteria

We included any peer-reviewed and published empirical article, written in English, that described a study conducted on human subjects deploying any kind of immersive VR device (eg, HMD, cave automatic VE), including 360° videos when used in a VR setup, focusing on any research question in the field of health promotion or primary prevention, from January 1, 2010, to September 16, 2024. We chose to limit the search to the last 14 years in order to generate a recent state-of-the-art overview of the field. We excluded studies conducted on nonhumans or focused on secondary or tertiary prevention interventions, such as psychotherapeutic treatments (eg, VR exposure therapy) and medical interventions (eg, rehabilitation), or specialized educational programs unrelated to prevention (eg, skills improvement for health practitioners). Pilot studies were not excluded from this review because of their critical role in assessing the feasibility and acceptability of interventions that may inform future primary, secondary, or tertiary prevention efforts. We excluded studies using the term “virtual reality” that described computer-based VEs involving a virtual world (eg, Second Life) or computer-related or motion-sensing devices (eg, Kinect, joystick) when they were associated with a nonimmersive VR setup (eg, non-VR video or serious game). We also used the population-concept-context framework to define our inclusion criteria. The population includes adolescents, young adults, and specific populations at risk for health issues (eg, individuals with anxiety or those at risk for substance use). The concept focuses on the application of VR technology to promote health behaviors, enhance knowledge, and improve emotional well-being. The context refers to contextual factors including the environments where VR interventions are delivered, such as schools, community centers, or health care facilities.

### Information Sources and Search Process

We searched 3 databases from January 1, 2010, until September 16, 2024 (PubMed and PsycINFO). For each database, we combined 2 sets of keywords; the first set focused on health promotion and prevention psychology. For PubMed, the search strings were (“health prevention” OR “health promotion” OR “health risk communication” OR “health communication” OR “preventive psychology” OR “behavior change” OR “attitude change”) AND (“virtual reality” OR “immersive virtual reality” OR “immersive virtual environment”). For PsycINFO, the search strings were (“health prevention” OR “health promotion” OR “health risk communication” OR “health communication” OR “preventive psychology” OR “behavior change” OR “attitude change”) AND (“virtual reality” OR “immersive virtual reality” OR “immersive virtual environment”).

### Selection of Sources of Evidence

Studies that did not employ VR technology, were not peer-reviewed, were reviews or meta-analyses, or lacked empirical data were excluded from the review. The screening process was conducted in 2 stages to enhance the rigor of the selection. In the first stage, titles and abstracts of the identified studies were reviewed to determine their relevance based on the inclusion criteria. This initial screening allowed the authors to eliminate studies that were clearly outside the scope of the review. In the second stage, full-text articles of the remaining studies were assessed to confirm their eligibility for inclusion. The extraction process was conducted independently by multiple reviewers to enhance reliability and minimize bias. Any discrepancies in data extraction were resolved through discussion and consensus among the reviewers. This meticulous approach to data extraction allowed the authors to synthesize findings across studies effectively and draw meaningful conclusions regarding the efficacy and feasibility of VR interventions in health promotion and primary prevention.

### Data Charting Process

The data charting process involved collecting information on study characteristics, intervention details, measured outcomes, user experience, type of materials, and sample characteristics (see Multimedia Appendix 1). Of note, approximately 63% of the studies included in the review were categorized as pilot or feasibility studies. We also recorded the type of VR technology used (eg, immersive headsets, desktop VR), the duration of the intervention, and the focus of the VR content (eg, health education, behavior change). On average, participants spent approximately 12.8 (SD 11.1) minutes using VR. We focused on health-related outcomes such as knowledge acquisition, behavioral intentions, and psychological well-being. User experience was assessed through qualitative data that provided insights into participants’ enjoyment, ease of use, and perceived effectiveness of the VR interventions. Many studies found that participants found the VR experience both enjoyable and engaging, which in turn led to higher participation rates compared to non-VR interventions.

### Data Items

Primary variables included study characteristics such as authorship, year of publication, study design, and sample size, which provided context for the research findings. Participant demographics, including age, gender, and health status, were also collected to understand the populations included in the studies. Intervention details were documented, focusing on the type of VR technology used, the duration of the intervention, and the specific health issues addressed. Measured outcomes were categorized into primary outcomes, such as knowledge acquisition and behavioral intentions, and secondary outcomes, including user engagement and satisfaction. User experience data were collected to assess participants’ enjoyment, ease of use, and any challenges encountered during the VR interventions. In addition, limitations of the studies were noted, including issues such as small sample sizes and methodological limitations, which are critical for contextualizing the findings. It is important to note that while immersion and presence are key concepts in understanding the effectiveness of VR, these variables were not measured consistently across studies, which may affect the interpretation of results. The data elements collected were intended to provide a structured review of the existing literature, as well as identify trends, gaps, and implications for future research in the field of VR-based health interventions.

### Critical Appraisal of Individual Sources of Evidence

We found that approximately 37% of the included studies were pilot or feasibility studies. These studies primarily focused on evaluating the usability and acceptability of VR interventions, which are critical for assessing the feasibility of larger-scale research. Although pilot studies provide valuable insights into user experiences and preliminary results, their small sample sizes and limited generalizability limit the ability to draw firm conclusions about the effectiveness of VR-based interventions. Mixed results have been found when comparing VR interventions to traditional methods, suggesting that VR does not always offer a clear advantage in achieving health outcomes. Key variables such as immersion and presence, which are critical to understanding how VR might influence health behaviors, have not been systematically evaluated. We found a lack of focus on larger, more diverse samples and aim to replicate existing studies to strengthen the evidence supporting the use of VR in health promotion efforts.

### Study Selection Procedure

All search results were stored in Zotero, an open-source reference manager, and duplicates were removed. Titles and abstracts were screened first, removing articles that clearly did not match eligibility criteria. Second, full texts of the remaining articles were downloaded to define final eligibility for inclusion. For each step, 2 reviewers conducted the screening independently and compared and discussed these discrepancies until a full consensus was reached.

### Data Extraction Process and Synthesis of Results

Data extraction was done by 1 reviewer, who extracted the following items from the included articles: (1) title and authors, (2) goal(s) of the study, (3) design of the study, (4) study sample characteristics, (5) VR device used, (6) main results, and (7) limitations reported by the authors. A second reviewer verified that all data were correctly extracted. Following the data extraction, we conducted a narrative analysis and synthesis of the results. Results and implications of the data extracted from the included studies were discussed by 2 reviewers in relation to the 3 research questions of the scoping review.

## Results

### Study Selection and Characteristics of Included Studies

The initial search identified 4295 unique articles, which were reduced to 51 eligible articles (see the PRISMA flowchart in Figure 1). Included studies were conducted in Asia (11 studies, 22%), Europe (18 studies, 35%), the Middle East (1 study, 2%), and North America (21 studies, 41%). The total sample size across all studies was 4647 participants, with an average of 91.1 participants per study. Study samples included slightly more women, with 2651 women (53%) and 1958 men (42.7%). The mean age of participants across the studies was 31.6 (SD 5.45) years. Studies primarily included adults, with 29 studies (57%) focused on adults, followed by 14 studies (28%) focused on adolescents, 7 studies (14%) focused on senior adults, and 1 study (2%) focused on children. Specific populations studied included students (7 studies, 29%), people with cognitive impairment (3 studies, 12%), and people with obesity (3 studies, 12%). Other populations studied included former smokers (1 study, 4%), NHS staff (1 study, 4%), parents (2 studies, 8%), smokers (2 studies, 8%), adults who had been in lockdown (1 study, 4%), and unvaccinated adults (1 study, 4%, see Figure 2 for details).

**Figure 1.**
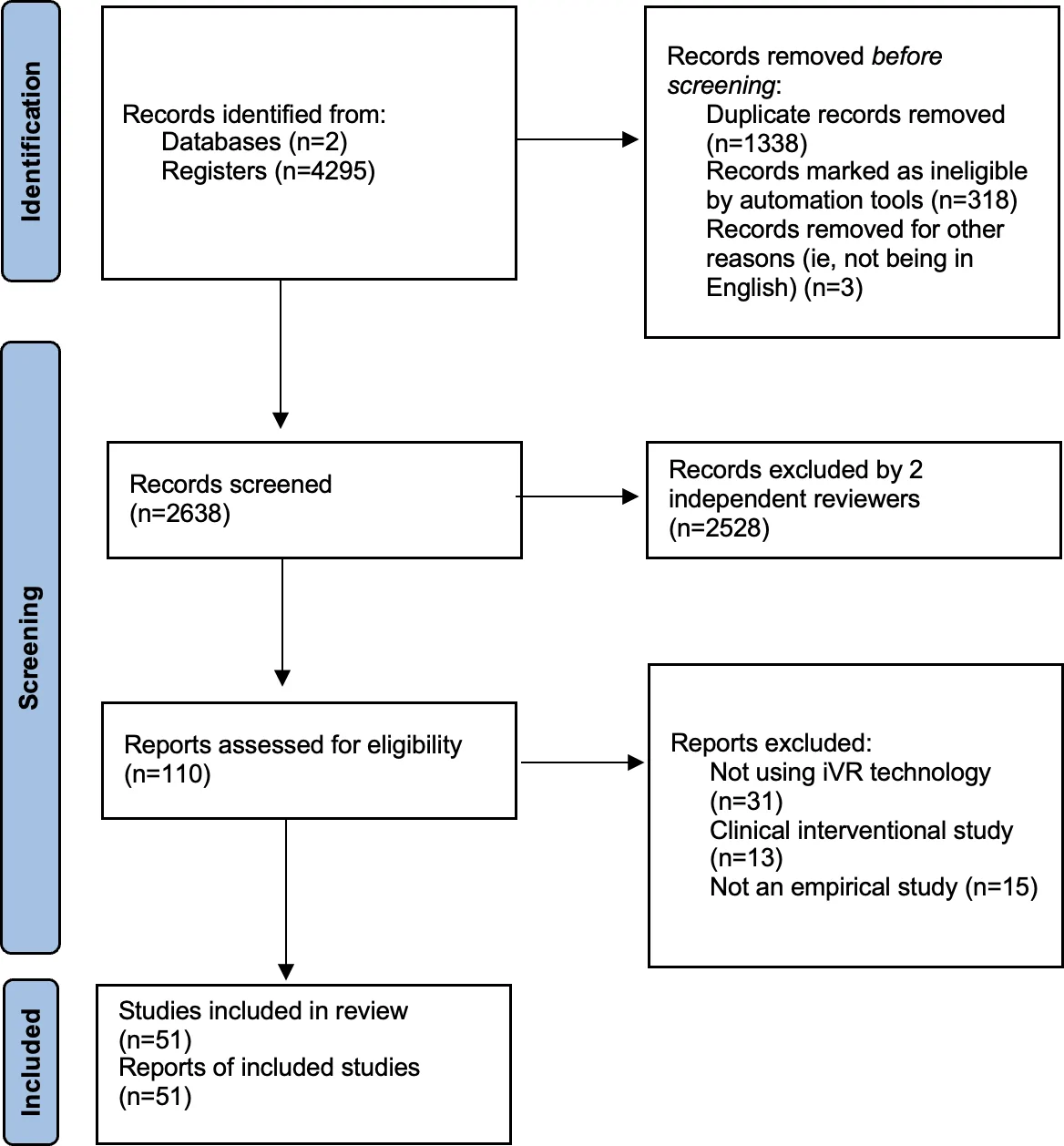
Flowchart of the study selection process following PRISMA guidelines. A total of 4295 articles were initially identified across 3 databases. After removing duplicates and applying eligibility criteria, 51 studies were included. iVR: immersive virtual reality; PRISMA: Preferred Reporting Items for Systematic Reviews and Meta-analyses.

**Figure 2.**
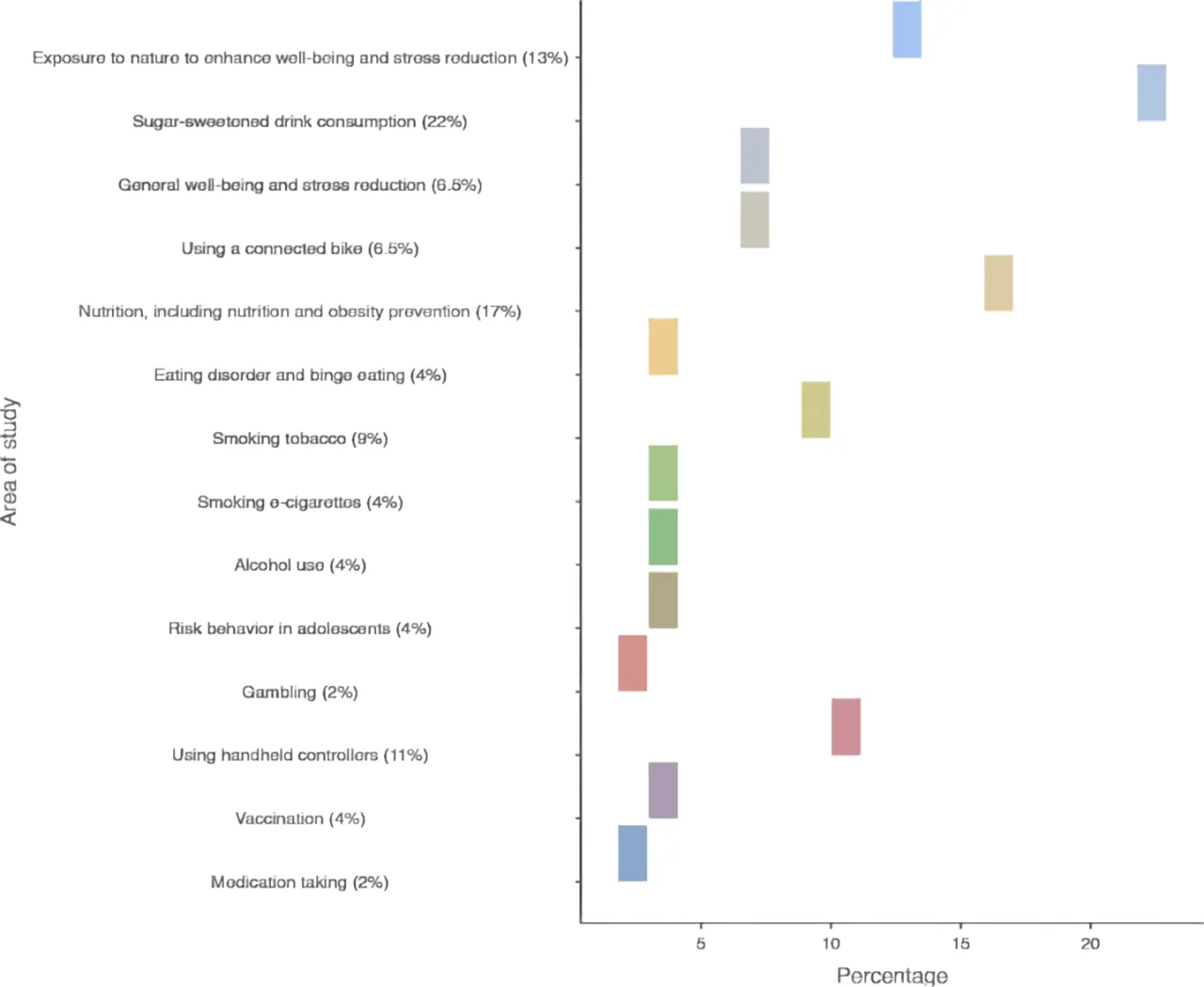
Overview of key outcomes from the intervention studies.

### Characteristics of Sources of Evidence

The 51 included studies focused on various health-related topics (Table 1), the most predominant ones being nutrition (17%) and risky behaviors (4%). All studies used HMD, except for Lemieux et al [25], where the device used was not mentioned. HMDs were mainly Oculus (Quest, Go, or Rift, 24%), HTC Vive (17%), or Samsung Gear VR (15%). Almost half of the studies (43%) were coupled with 1 or 2 handheld controllers. Most studies (56%) included an active interaction with the VE by using 1 or 2 handheld controllers or the bodysuit to interact with the VE. About 49% of VR exposure lasted a maximum of 10 minutes, including 22% of studies with under 5 minutes of VR exposure. We estimated an average time of 12.8 (SD 11.1) minutes spent using VR, according to the information given in the articles.

**Table 1.** Characteristics of sources of evidence.

Area of study	Studies
Nutrition, including nutrition and obesity prevention (17%)	Blom et al [26]; Isgin-Atici et al [27]; Ledoux et al [28]; Marcum et al [29]; McBride et al [30]; Persky et al [31,32]; Verhulst et al [33]
Eating disorder and binge eating (5%)	Ferrer-Garcia et al [34]; Lemieux et al [25]
Sugar-sweetened drink consumption (10%)	Blom et al [26]; Ledoux et al [28]; Marcum et al [29]; McBride et al [30]
Smoking tobacco (8%)	Borrelli et al [35]; Ferrer-García et al [36]; García-Rodríguez et al [37]; Bonneterre et al [17]
Smoking e-cigarettes (5%)	Weser et al [38,39]
Alcohol use (5%)	Guldager et al [40]; Ma [41]
Risk behavior in adolescents (4%)	Hadley et al [42,43]
Gambling (2%)	Detez et al [44]
Exposure to nature to enhance well-being/stress reduction (14%)	Alyan et al [45]; Beverly et al [46]; Brimelow et al [47,48]; Browning et al [49]; Calogiuri et al [50]
General well-being/stress reduction (10%)	Afifi et al [51]; Adhyaru et al [52]; Kim et al [53]; Riva et al [54]; Ko et al [55]; Kiper et al [56]
Using handheld controllers (7%)	Eisapour et al [57]; Fang and Huang [58]; Farič et al [59]
Using a connected bike (7%)	Bird et al [60]; Zeng et al [61,62]
Vaccination (4%)	Mottelson et al [63]; Nowak et al [64]
Medication-taking (2%)	Niki et al [65]

### Results of Individual Sources of Evidence: Detailed Results

#### Main Identified Research Goals

We identified three main goals for using VR: (1) as a tool to deliver an intervention, with 35 articles focusing on either (1a) pilot testing or testing the feasibility of using VR materials or procedures or (1b) using VR to deliver an actual intervention (eg, skills learning, comparing VR vs other intervention modalities) to test its relative efficacy; (2) as a tool to address fundamental research questions, with 6 studies aimed at recreating ecological settings to address physiological and psychological changes when exposed to certain situations (eg, cravings elicitation); or (3) as an assessment tool, with 5 studies investigating food choices with a food buffet created in VR.

#### Pilot Studies: Ensuring Usability and Enjoyability

Many studies included in the scoping review were pilot or feasibility studies (about 37%, Table 2) from which we distinguished two main purposes: (1) testing VR usability for future research and seeing how target outcomes are impacted and (2) assessing users’ experience with VR. First, researchers found that the use of VR in their methods was rather relevant and reached multiple target outcomes such as reducing stress using a short exposure to nature in VR [45-48,51,52,55], even though exposure durations were relatively short (3-10 minutes). The use of VR was also useful to enhance participants’ physical and cognitive activity [66,67]. Finally, some studies were focused on prevention and the major advantage of VR use is its ability to involve participants directly in the preventive message, for example through gaming [68] or skill practice (eg, refusing peer pressure to vape [38]). This resulted in improved knowledge on health topics (eg, on smoking in [69]) and intentions to check for diseases (eg, [20]). It also helped to deliver information in a more traditional preventive way (eg, exposure to a preventive video in an HMD in [35] or a FestLab in [40]). Overall, pilot and feasibility studies, even if conducted on small samples, found VR to be enjoyed and accepted by participants, as well as useful and feasible, and found that it impacted target outcomes (eg, enhanced well-being, increased knowledge). These results occurred whether participants only had a one-time exposure (eg, [53,69]) or sessions over a few weeks (eg, [48]) and were found to be sustained at follow-up when measured (eg, participants reduced their tobacco intake over the month following their participation [35]).

**Table 2.** Summary of articles and their classification within the scoping review.

Category and study	Descriptives	iVR^a^ details	Objective(s)	Study design	Main conclusions
**1a: Pilot or feasibility studies**					
Adhyaru and Kemp [52]	n=39; mean age 36.6 (SD 10.3) years; 82% women; health care workers	HMD^b^ (Oculus Go); 10 minutes	Explore if exposure to nature in iVR can help health care workers destress at work.	Before-after exposure; within-subject	iVR reduced anxiety, anger, and heart rate, and enhanced happiness and relaxation.
Afifi et al [51]	n=50 older adults with cognitive impairments and their family members	Immersive VR^c^ system	Assess whether iVR improves quality of life and social interaction for older adults and their family members.	Feasibility study with pre-post assessments	VR improved social interaction and quality of life for both older adults and their families.
Alyan et al [45]	n=20; mean age 21.8 (SD 2.2) years; 50% women; students	HMD (HTC Vive); 5 minutes	Use iVR to reduce stress via a virtual walk in nature.	2 (environment: realistic vs dreamlike); between-subject	iVR reduced stress and enhanced mental well-being.
Beverly et al [46]	n=102; 72% women; health care workers	HMD (Oculus Go/Pico G2); 3 minutes	Explore if cinematic iVR can reduce stress in health care workers.	Before-after exposure; within-subject	iVR reduced stress, independently of previous iVR use or job type.
Bonneterre et al [17]	n=121; mean age 19.6 years; 82.5% female; university students	Sensiks Immersive VR system	Evaluate the impact of VR on memorization, attitudes, and craving responses to anti-tobacco posters.	Randomized controlled trial	VR enhanced memorization of prevention messages.
Borelli et al [35]	n=23; mean age 49.8 (SD 13.3) years; 22% women; adult smokers	HMD (Knoxlabs V2 cardboard); 5 minutes	Examine the feasibility and impact of a smoking cessation intervention during dental cleaning.	2 (video type: smoker ready/not ready to quit) × 3 (time: pre/post/follow-up); within-subject	Feasible and accepted by both smokers and dental care providers.
**1b: Interventions**					
Ahn [5]	n=73; mean age 20.8 (SD 1.1) years; 82% women; students	HMD (NM); 2 minutes	Test efficacy of preventive messages on sugar and sweetened beverage consumption via avatar embodiment.	2 (pamphlet only vs pamphlet plus iVR) × 2 (tailoring: others vs self) × 3 (time: pre/post/follow-up); between-subject	iVR heightened intentions to limit sugar and sweetened beverage consumption; effects were present at follow-up.
Blom et al [26]	n=99; mean age 30.7 (SD 10.9) years; 60% women; general population	HMD (HTC Vive); ≥3 minutes	Study purchase behaviors in an iVR supermarket.	2 (nudge vs control) × 2 (time pressure: 3 minutes vs no pressure); between-subject	iVR revealed changes in healthy food purchases based on nudge type.
**2: Fundamental research**					
Chittaro et al [70]	n=105; mean age 21.49 (SD 2.43) years; 90.5% women; students	HMD (Sony HMZ-T1); 5 minutes	Investigate links between iVR and persuasion theory, including inducing mortality salience.	2 (environment: iVR park vs cemetery); between-subject	iVR elicited mortality salience, impacted attitudes, and induced greater physiological reactions than traditional mortality salience manipulations.
Ferrer-Garcia et al [36]	n=25; mean age 29.7 (SD 13.4) years; 32% women; smokers	HMD (5DT HMD 800); time not mentioned	Assess iVR’s ability to produce cravings toward tobacco smoking.	Before-during exposure to smoking cues	iVR created cravings, correlated with presence.
**3: Assessment tool**					
Isgin-Atici et al [27]	n=73; mean age 22.2 (SD 4.1) years; 56% women; students	HMD (HTC Vive); 5‐25 minutes	Evaluate ease of use and efficiency of a virtual cafeteria.	2 (groups: iVR novices vs experienced); between-subject	iVR was user-friendly and effective regardless of prior VR experience.
Marcum et al [29]	n=221; mean age 38 (SD 5.6) years; 100% women; mothers with obesity	HMD; time not mentioned	Examine microbehaviors influencing food selection in an iVR buffet.	3 (conditions: food safety control vs behavioral risk information vs family-based risk information); between-subject	iVR enabled dynamic assessment of food choice behaviors.

a iVR: immersive virtual reality.

b HMD: head-mounted display.

c VR: virtual reality.

Second, most participants found VR enjoyable and fun [59,68] and quite easy to use [52]; some were asked to complete a short tutorial [27]. Even older adults were able to manipulate handheld controllers [57], but 1 study reported that the HMD is sometimes heavy for their neck to lift (1 participant dropped out because of this reason [52]). It is worth noting that some of these studies [20,59] involved the targeted population in co-designing the intervention in previous pilot studies, hence not only explicitly ensuring usability [57] but also enhancing users’ satisfaction with the intervention. Co-designing an intervention with the targeted population and conducting a first pilot study on a small sample (eg, 12/33) can improve the level of satisfaction and usability of the intervention prototype, albeit ultimate user satisfaction can only be assessed following full-scale deployment of the intervention.

#### Relative Efficacy of VR Interventions

Interventions (39% [20/51] of the studies included in the review) using VR focused on several targets such as enhancing well-being by simulating a walk in nature (while remaining seated [49,54] or walking on a treadmill [50]) or skill learning and practice on various health topics [40,42]. Some studies were interested in delivering preventive content [40,41,71], other studies used VR’s ability to create standardized conditions to test theoretical frameworks (eg, nudge and time pressure on healthy food choice [26,40]), while still others used VR to embody a specific character in order to impact health outcomes [5,33,56].

The key element of most studies included in this group is that they often compared the use of VR with other modalities to deliver an intervention; for example, delivering preventive information in VR versus a 2D screen (eg, [41,54]) or without the use of specific technology (eg, live role-playing with an instructor [64], reading a pamphlet, [61]). Some studies also compared different depths of immersion [50,60,62].

When comparing the relative efficacy of VR with other modalities, mixed results were found. For example, even though participants exercising using VR experienced an attentional shift from exercising, meaning that individuals were usually distracted and entertained by the VR setting, leading them to actually enjoy physical exercise, it was not always sufficient to obtain greater physical involvement when compared to nonimmersive physical activities [25,58,60,62]. However, some studies found no difference in outcomes between the use of VR and 2D screens [54,71], and other studies even found that a virtual walk remained less efficient than a real walk in nature for mood enhancement [49]. Some studies, using VR only, also found no impact of VR prevention interventions on target outcomes (eg, no change in physical self-perception when using VR to prevent eating disorders [39], no increased knowledge on alcohol [40]). Still, we note that VR was a great tool to induce changes in knowledge and intentions to adopt a behavior (eg, vaccination intention [61], smoking e-cigarettes [44]) and for skill practice [42].

A few recent studies [44,54] investigated the use of VR outside of the laboratory, recruiting participants who own VR devices at home. Portable VR devices have become more affordable, resulting in individuals being able to use them potentially anywhere and be autonomously engaged with VR-based interventions. Furthermore, both studies resulted in an improvement in the target outcomes (reduction of psychological distress [54], increase in vaccination [44]).

Overall, VR is impactful; it can create precise and standardized experimental situations (eg, embodying an obese or weight-gaining avatar [5,33]), and it is especially practical for skill practice and sometimes for physical activity. VR-based interventions have shown a higher degree of attendance in intervention sessions (ie, adherence) than the same intervention done without the use of VR [64]. However, when VR is only used to deliver information without leveraging its specific characteristics, such as immersivity and active use of the device (ie, interacting with the VE via a game [61]), it has often been found to have similar efficacy as more traditional ways to deliver information (eg, 2D screens).

#### VR to Address Fundamental Health Research Questions: A Tool to Recreate Ecological Settings in the Lab

VR can recreate real-life situations in laboratories and has been used across different domains, such as gambling [36], tobacco cravings [28,37], and food cravings [34,70], as well as for mimicking specific situations inducing certain psychological states, such as mortality salience (eg, [29]). In all studies, exposure to specific cues (eg, food items, cemetery, individuals smoking) or situations (eg, being in a pub, gambling on a slot machine) elicited both physiological (eg, increased heart rate, arousal) and psychological (eg, self-reported craving) changes, whether individuals were actively (ie, interacting with the VE) or passively (ie, watching visual content) using the VR device, suggesting that the highly immersive characteristics of VR are effective at eliciting an emotional response.

However, only 1 study compared eliciting cravings using VR versus other types of devices [34], indicating VR is not better suited to trigger a craving response than 2D pictures. It might be possible that this null effect was due to the passive use of VR in this specific study, as interacting with a cue in VR has been found to enhance cravings [28].

#### VR as an Assessment Tool in Health-Related Interventions

A total of 5 studies used VR as an assessment tool in the field of nutrition by recreating a virtual buffet displaying food [27,30-32,65], where participants’ task was to collect a plate of food. Participants found the VR food buffet easy to use, independently of whether they already used a VR device in the past [27]. In this context, VR allows researchers to study precisely how many items and types of food were selected and in which quantity, enabling them to calculate the total calories contained in each plate more easily. It also helped to display to participants a standardized food buffet with diverse food items without constraints from a real food buffet (eg, expiration dates, flexibility in food types, reduced costs).

### Study Limitations

#### The Necessity to Adapt the Use of VR to Experimental Needs

The use of VR, whether for applied or fundamental research, has shown some limitations, mainly related to the study methodology and VR technology itself (eg, cybersickness, notably in [50]). First, a majority of included studies suffered from either small sample sizes (eg, 10 participants in [65], 6 in [57]) or nonrepresentative samples (eg, students in [29], healthy and active young individuals in [58]), limiting the validity and generalizability of results. Second, the quality of the experimental designs was sometimes limited (eg, semiexperimental design with pre-post comparisons) because of a lack of a proper control condition or not conducting a rigorous randomized controlled trial [20,46]. Short-term follow-up or the lack of a follow-up altogether was also mentioned as a limiting factor in numerous studies [41,42].

#### Lack of Systematic Assessment of VR’s Main Characteristics: Presence, Immersion, and Cybersickness

VR’s effects, especially persuasive effects, seem to come from its ability to enhance presence, which is the feeling of being there during a VR experience. Hence, participants act similarly to real life in the VE because they are fully immersed in their interaction with it. The level of presence experienced by users can impact targeted variables in the intervention; participants who felt more present in the VE showed stronger positive effects on persuasion-related outcomes (eg, attitudes toward vaccination and intention to get vaccinated [61]; higher presence resulted in more reported cravings for tobacco in [37]). However, presence is rarely measured as a moderator or covariate across studies despite its potential impact on outcomes. The same applies to immersion, which was not measured across studies, despite studies often comparing different intervention modalities of varying degrees of immersion (eg, VR versus 2D screen). VR is not the only technology able to generate presence; narrative, videos, or nonimmersive VR can too [14]. Not measuring immersion or presence across different modalities limits the understanding of VR’s role in driving effects on the target outcomes.

Finally, cybersickness was rarely measured across studies despite its potential negative effect on user experience and, in turn, target outcomes. Some studies, notably the ones focusing on physical activity, measured cybersickness and found that it can completely erase the positive effects of using VR (eg, walking on a treadmill while wearing a VR device led to cybersickness, which diminished the positive effects of being exposed to nature compared to the other condition, [50]). Participants who felt symptoms of cybersickness believed that it impacted their experience [59], sometimes to the point they had to drop out of the experiment [62].

## Discussion

### Principal Findings

This scoping review identified 51 studies published over the past 14 years that explored the use of VR in health promotion and prevention psychology. Our findings revealed three primary applications of VR: (1) as a tool to deliver interventions, either in feasibility testing or actual implementation; (2) as a means to address fundamental research questions; and (3) as an assessment tool for health-related outcomes. Although VR shows significant promise in creating immersive and engaging interventions, our review highlights the variability in effectiveness and common challenges such as small sample sizes, short follow-up periods, and limited methodological standardization.

VR technology use for health promotion and prevention research is relatively recent, with studies in this review indicating its potential as a promising tool to deliver and assess interventions. For instance, VR was effective in simulating realistic scenarios to engage participants in skills-based learning and decision-making tasks, such as risk-reduction behaviors [42,61]. VR allows researchers to create safe, ecological, and standardized VEs in which it is possible to deliver and evaluate health promotion and preventive interventions [42]; recreate situations or environments that can elicit strong emotional, physiological, behavioral, or psychological responses (eg, mortality salience [29]); and assess outcomes (eg, cravings, food choices) with a multimeasure approach included in VR technologies (eg, psychological, physiological, and behavioral measures). This scoping review identified 51 studies concerning the use of VR technology in the field of health promotion and prevention psychology published within the past 14 years. We mapped (1) the goals and research questions addressed through the use of VR in this field, (2) its effects in the identified areas, and (3) its main challenges or limitations. We identified three main applications of VR in this field: (1) as a tool to deliver an intervention, either (1a) pilot or feasibility testing VR materials or procedures or (1b) using VR to deliver an actual intervention (eg, skills learning, comparing VR vs other intervention modalities) to test its relative efficacy; (2) as a tool to address fundamental research questions; and (3) as an assessment tool.

### Comparison to Prior Work

Due to the relative novelty of VR in this field, only 51 eligible studies were published in the past 14 years. Research so far has mostly focused on feasibility or pilot studies, aimed at testing the ability of VR to be integrated into interventions [69], with a minority of studies focusing on answering fundamental research questions through the use of VR [28]. Most studies employed semiexperimental designs without a control or comparison group and often had a short or no follow-up, limiting the validity and generalizability of results. Studies also included relatively small samples and were often nonrepresentative of the general population (eg, students). However, as the use of VR in the field of health promotion and prevention is still in its infancy, it appears natural to see a stronger focus on pilot or feasibility studies in the published literature.

### Strength and Limitations of the Scoping Review

When considering whether VR is effective in health prevention, it should first be noted that the effectiveness of VR interventions was variable. For instance, while some studies indicated that VR could enhance user engagement and motivation [59], others found no significant differences in outcomes compared to traditional methods [71]. This highlights the need for further research to clarify the conditions under which VR is most effective. This scoping review showed that sometimes VR use is not systematically more effective in achieving target outcomes than its nonimmersive equivalents [41,54,71]; we supposed that to be more effective, VR should be used for its specific immersive characteristics, such as gamification or embodiment, which directly involve the user. For example, skills practice in VR was more effective than role-playing in real life to learn about risk behaviors and ways to avoid them (eg, buying condoms for safer sex) due to VR scenarios’ ability to recreate a situation that is realistic, induce emotional changes in the user as the scenario goes on, and finally, make the user have a real first-person experience [42]. Similar results appeared in [61], in which VR was used to represent a vaccination intervention to stop flu spread (ie, participants used handheld controllers to actively send immune cells to prevent flu transmission), whereas in other conditions, participants were just passively watching (a video on a 2D screen or a pamphlet).

It is important to consider the limitations of this review when interpreting the findings. First, as the use of VR in health promotion and prevention psychology is a relatively recent phenomenon, our literature search focused on the last 14 years (2010‐2024). This resulted in the inclusion of 51 eligible articles, which may have excluded earlier or less accessible studies. However, the majority of included studies (63%) were published between 2020 and 2024, reflecting the increasing affordability and accessibility of VR technology for research in recent years. Therefore, the likelihood of missing pivotal studies is low. Second, the search strategy did not include gray literature, which may have reduced the total number of eligible articles and introduced publication bias by excluding studies with nonsignificant or null results (the file drawer effect). To address this gap, future reviews should consider including gray literature to provide a more comprehensive overview of the field. Third, some studies lacked sufficient reporting of critical aspects such as sample characteristics (eg, size and demographics) and details of VR implementation (eg, exposure duration, type of VR technology used). This limited our ability to draw broad conclusions about the efficacy and applicability of VR in this area. Addressing these reporting gaps in future research will improve the comparability and quality of evidence in this rapidly evolving area of study. Fourth, although our literature search was updated during the initial revision, which was completed just a few weeks prior to this submission, we recognize that VR research is advancing rapidly. It is therefore possible that new studies may emerge shortly after the conclusion of our search period, which may influence the results of future reviews. To address this, future updates could consider conducting more frequent searches or establishing a continuous review process to ensure that all emerging data are included in real time. However, we are confident that this review accurately reflects the state of the literature as of our latest search.

### Perspectives and Future Research Directions

#### Standardization of Designs and Replication

Although the results of our scoping review suggest that VR has potential as a tool for health promotion, the field is still in its infancy. Many studies in this area are limited by small sample sizes, short follow-up periods, and inadequate experimental control. Replication is essential to strengthen the reliability and validity of these findings [72,73]. Replication of these studies in diverse populations and settings will help confirm the generalizability of the findings and identify any boundary conditions, such as differences in user demographics, technology exposure, or the specific health behaviors targeted [74,75]. In addition, replication can shed light on the mechanisms underlying the effectiveness of VR interventions, which may vary depending on the context and population studied. Therefore, further replication is essential not only to solidify current evidence, but also to ensure that VR interventions are applicable and effective across a wide range of health promotion and primary prevention efforts. Replicating existing results to increase the amount and quality of empirical evidence supporting the use and benefit of VR in this field is needed. For example, in this scoping review, we saw that individuals showed an increased knowledge regarding health-related topics [20,69] or changed their behavioral intentions [44,61] when exposed to a VR intervention. However, not all studies provided evidence to fully support these claims [47,63], in addition to the lack of any perspective on how long these effects last or if they are applicable to less specific populations. Therefore, a focus on study replication can strengthen the advancement of research in this field and at the same time prevent a replication crisis, as observed in other fields of behavioral sciences and medicine [76]. There is also a critical need for future research to employ longitudinal study designs. Long-term follow-up is particularly important in preventive psychology, where sustained behavior change and long-term health outcomes are key indicators of success.

#### VR vs Nonimmersive Apparatus

The effectiveness of VR compared to nonimmersive interventions, such as 2D presentations, remains controversial. Evidence from the studies included in this review showed mixed results. Although some studies reported that VR interventions increased engagement and enjoyment, others found no significant differences in outcomes compared to nonimmersive methods [59,71]. The immersive features of VR, such as gamification and embodiment, appear to be particularly effective in scenarios that require active user involvement. For example, participants who practiced risk-avoidance skills in VR showed better retention than those who used real-life role-playing [42]. Similarly, the use of VR in interactive scenarios, such as vaccination education, showed higher levels of engagement than passive modalities such as 2D videos or pamphlets [61]. However, studies have also shown that VR does not always outperform traditional methods in terms of physical activity or knowledge acquisition. This variability highlights the need for future research to clarify the specific contexts in which the immersive qualities of VR are most effective. Systematic assessment of key mechanisms such as presence and immersion could help determine whether VR’s effectiveness is primarily due to its immersive nature or to other factors such as interactivity or novelty.

Assessing presence and immersion is crucial for understanding the mechanisms underlying VR and its effects [14]. Evaluating the feeling of presence helps determine the extent to which participants are psychologically immersed in VEs and allows for the identification and correction of potential errors in the VE that could influence presence and, consequently, the effectiveness of VR-based interventions or content. Additionally, addressing such errors can prevent cybersickness and ensure the smooth execution of experiments [50]. Measuring presence and immersion provides valuable insights into individuals’ capacity to engage with VR compared to nonimmersive interventions and helps identify how these factors correlate with target outcomes.

#### Set Up for Success

Conducting feasibility or pilot studies to test the VR procedure and VEs is recommended. As shown in the scoping review, evaluating the enjoyability, usability, and safety of the procedure can be very helpful. Finally, co-designing the VR-based intervention with participants from the targeted population can enhance the relevance, validity, and user experience with the intervention itself. Cocreating a procedure with participants could induce a bias in their judgment, making them judge the intervention more positively than it actually is. Pilot testing with different groups of participants is recommended to validate the final design.

#### Make It Simple and Clear for Participants

VR studies are attractive to participants (eg, higher attendance for intervention sessions than the non-VR condition in [19,42,77]), but they can be complex to follow all the way through (ie, risk of cognitive overload, fatigue [78]). When designing studies using VR, keeping them as simple and short as possible will minimize participant burden and fatigue. It is also highly possible that most participants have never experienced VR before, so making sure they understand how to move and interact with the environment at first is necessary. If possible, we recommend doing a short tutorial on how to use the controllers or putting the participant in a tutorial VE before the experimental procedure. The participants can then fully concentrate on what is happening in the VR rather than think about how to interact with the VE.

### Conclusion

This scoping review provides an overview of VR’s emerging role in health promotion and prevention psychology, highlighting its potential to create immersive and engaging interventions. Although VR has shown promise in delivering health interventions and answering fundamental research questions, its effectiveness remains variable, and many studies are limited by methodological constraints. Future research should prioritize replication, longitudinal designs, and standardized methodologies to strengthen the evidence base and realize the full potential of VR in this field.

## Supplementary material

10.2196/49923 Multimedia Appendix 1 Summary of Studies Using Virtual Reality for Health-Related Behavioral Interventions.

10.2196/49923 Checklist 1 PRISMA-ScR checklist.

## Abbreviations

HMD: head-mounted display
PRISMA-ScR: Preferred Reporting Items for Systematic Reviews and Meta-analyses extension for Scoping Reviews
VE: virtual environment
VR: virtual reality
