# Supplementary material for: Immersive Virtual Reality for Health Promotion and Primary Prevention in Psychology: Scoping Review
Source: JMIR XR Spat Comput. 2025 Jan 20;2:e49923. doi: 10.2196/49923 (PMC13711389; doi:10.2196/49923)
Supplement: Supplementary file 2 [file xr-v2-e49923-s001.docx]

**Appendix B: Summary of Studies Using Virtual Reality for Health-Related Behavioral Interventions**

| **Title** | **Authors** | **Topic** | **Year of Publication** | **Continent** | **Country** | **Nb_included_studies** | **% ou nb women** | **nb women** | **%women** | **nb non-binary** | **% non-binaire** | **% ou nb men** | **nb men** | **%men** | **nb de participants** | **age moyen des participants** | **mean age** | **standard deviation age** | **Population** | **Population_Specific** | **Population_specific_old** | **type VR (HMD, etc)** | **VR Model (Oculus, etc)** | **VR Accessory (controllers, etc)** | **Time spent in VR** | **Time_category** | **passive/active** | **Keyword** |
| --- | --- | --- | --- | --- | --- | --- | --- | --- | --- | --- | --- | --- | --- | --- | --- | --- | --- | --- | --- | --- | --- | --- | --- | --- | --- | --- | --- | --- |
| VR as a tool to promote wellbeing in the workplace | Adhyaru et Kemp | well-being | 2022 | Europe | UK | 1 | 32 women (83,8%) | 32 | 82.05 |  | 0 | 7 men (16,2%) | 7 | 17.95 | 39 | M=36,61 ans, SD= 10,26 allant de 25 à 60 ans | 36.61 | 10.26 | Adults | NHS staff | NHS staff | HMD | Oculus Go | 1 controller | 10 min | 10 to 15 | Passive | wellbeing promotion in NHS staff |
| Incorporating Immersive Virtual Environments in Health Promotion Campaigns: A Construal Level Theory Approach | Ahn | SSB consumption | 2015 | North America | US | 1 | 60 women | 60 | 82.19 |  | 0 | 13 men | 13 | 17.81 | 73 | M=20,83 (SD=1,05) | 20.83 | 1.05 | Adolescents |  | undergraduate student | HMD | unspecified | none | 2 min | <5 | Passive | SSB drink prevention and reduction |
| The Influence of Virtual Forest Walk on Physiological and Psychological Responses | Alyan | exposure to nature | 2021 | Asia | Malaysia | 1 | 10 women | 10 | 50.00 |  | 0 | 10 men | 10 | 50.00 | 20 | M=21,8 (SD= 2,2) | 21.8 | 2.2 | Adults | Students | healthy university student | HMD | HTC Vive | touchpad | 5 min | 5 to 10 | Passive | Stress reduction through forest walk |
| A tranquil virtual reality experience to reduce subjective stress among COVID-19 frontline healthcare workers | Beverly | exposure to nature | 2022 | North America | US | 1 | 73 women (71,6%) | 73 | 71.57 |  | 0 | 29 men | 29 | 28.43 | 102 | 49 (48%) between 25 ans 34 years old |  |  | Adults | healthcare workers | healthcare worker | HMD | Oculus Go ou Pico G2 4K | none | 3 min | <5 | Passive | Stress reduction in health workers |
| Ready Exerciser One: Effects of music and virtual reality on cycle ergometer exercise | Bird | physical activity | 2021 | Europe | UK | 1 | 10 women | 10 | 41.67 |  | 0 | 14 men | 14 | 58.33 | 24 | M=26,7 (SD=4,1) | 26.7 | 4.1 | Adults |  | recreationnaly active adult | HMD | Samsung Gear VR + Samsung S8 | VirZOOM bike Controller | 12 min x2 | 10 to 15 | Passive | Effet of music and VR on cycle ergometer exercice |
| Under pressure: Nudging increases healthy food choice in a virtual reality supermarket, irrespective of system 1 reasoning | Blom | Nutrition | 2021 | Europe | Netherlands | 1 | 59 women | 59 | 59.60 |  | 0 | 40 men | 40 | 40.40 | 99 | M=30,70, SD=10,90 | 30.7 | 10.9 | Adults |  | Adults | HMD | HTC Vive | 2 controllers | 3 min minimum | <5 | Active | Nudging healthy food choice in virtual supermarket |
| Delivery of a smoking cessation induction intervention via virtual reality headset during a dental cleaning | Borelli | Smoking | 2019 | North America | US | 1 | 5 (22%) women | 5 | 21.74 |  | 0 | 18 (78%) men | 18 | 78.26 | 23 | M=49,8, SD = 13,3 | 49.8 | 13.3 | Adults | Smokers | Adults smokers | HMD | Knoxlabs V2 cardboard | none | 5 min | 5 to 10 | Passive | Smoking prevention while dental cleaning |
| Preliminary Research: Virtual Reality in Residential Aged Care to Reduce Apathy and Improve Mood | Brimelow | exposure to nature | 2020 | Asia | Australia | 1 | 9 women | 9 | 69.23 |  | 0 | 4 men | 4 | 30.77 | 13 | M=82, SD=8 | 82 | 8 | Senior Adults | Cognitive Impairment | Adults in Residential aged care, all with dementia and different levels of cognitive impairment | HMD | Samsung Gear VR + Samsung S7 | none | 4-5 min | <5 | Passive | Reduce apathy and mood via relaxing VR videos |
| Feasibility of Group-Based Multiple Virtual Reality Sessions to Reduce Behavioral and Psychological Symptoms in Persons Living in Residential Aged Care | Brimelow | exposure to nature | 2021 | Asia | Australia | 1 | 15 women (60%) | 15 | 60.00 |  | 0 | 10 men | 10 | 40.00 | 25 | unspecified |  |  | Senior Adults | Cognitive Impairment | Adults in Residential aged care, all with dementia and different levels of cognitive impairment | HMD | Samsung Gear VR + Samsung S8 | none | 10 min | 10 to 15 | Passive | VR intervention to reduce apathy and mood in RCA |
| Can Simulated Nature Support Mental Health? Comparing Short, Single-Doses of 360-Degree Nature Videos in Virtual Reality With the Outdoors | Browning | exposure to nature | 2020 | North America | US | 1 | 39 women | 39 | 47.56 |  | 0 | 43 men | 43 | 52.44 | 82 | M=20, SD=1.2 | 20 | 1.2 | Adolescents |  | undergraduate student | HMD | Samsung Gear VR + Samsung Galaxy Note 5 | none | 6 min | 5 to 10 | Passive | Nature irl vs IVR nature for mental well-being |
| Experiencing Nature through Immersive Virtual Environments: Environmental Perceptions, Physical Engagement, and Affective Responses during a Simulated Nature Walk | Caloguiri | exposure to nature | 2018 | Europe | Norway | 1 | 12 women | 12 | 46.15 |  | 0 | 14 men | 14 | 53.85 | 26 | M=26, SD=8 | 26 | 8 | Adults |  | adults aged 20-45 years | HMD | Samsung Gear VR + Samsung S7 | | 10 min | 10 to 15 | Passive | Nature walk irl vs VR effect on well-being |
| Mortality Salience in Virtual Reality Experiences and its Effects on Users’ Attitudes towards Risk | Chittaro | mortality salience | 2017 | Europe | Italy | 1 | 95 women | 95 | 90.48 |  | 0 | 10 men | 10 | 9.52 | 105 | M=21.47 (SD= 2.78) cemetery VE group; M=21.50 (SD=2.08) | 21.47 | 2.78 | Adolescents |  | graduate and undergraduate students | HMD | Sony HMZ-T1 + 3-DOF sensor | 1 controller | 5 min | 5 to 10 | Passive | VR mortality salience and its impact on attitudes towards risk |
| A Psychophysiological and Behavioural Study of Slot Machine Near‑Misses Using Immersive Virtual Reality | Detez | gambling | 2019 | Asia | Australia | 1 | 25 women | 25 | 49.02 |  | 0 | 26 men | 26 | 50.98 | 51 | M= 23.59, SD= 7,79 | 23.59 | 7.79 | Adults |  |  | HMD | HTC Vive | 1 controller | 15-20 min | 15 to 20 | Active | Slot machine iVR for near misses to study psychophysiological and behavior |
| Participatory design and evaluation of virtual reality games to promote engagement in physical activity for people living with dementia | Eisapour | physical activity | 2020 | North America | Canada | 1 | 5 women | 5 | 83.33 |  | 0 | 1 men | 1 | 16.67 | 6 | M= 86.8, SD= 6.2 | 86.8 | 6.2 | Senior Adults | Cognitive Impairment | old adults with dementia | HMD | Oculus Rift CV1 | 2 controllers | 5 min | 5 to 10 | Active | Design of VR promote engagement in physical dementia |
| Comparison of the usability and flow experience of an exercise promotion virtual reality programme for different age groups | Fang et Huang | physical activity | 2021 | Asia | Taiwan | 1 | 45 women | 45 | 72.58 |  | 0 | 17 men | 17 | 27.42 | 62 | 19-75+ years old, but 48,4% de 19-28 years old |  |  | Adults |  | adults | HMD | HTC Vive | 2 controllers | 10 min max | 5 to 10 | Active | exercice promotion for different age group, VR vs IRL |
| A Virtual Reality Exergame to Engage Adolescents in Physical Activity: Mixed Methods Study Describing the Formative Intervention Development Process | Faric | physical activity | 2021 | Europe | UK | 1 | 17 women | 17 | 65.38 |  | 0 | 19 men | 19 | 73.08 | 26 | between 13 and 17, no more precision |  |  | Adolescents |  |  | HMD | HTC Vive | 2 controllers | unspecified |  | Active | creating exergame iVR for adolescents |
| Efficacy of Virtual Reality in Triggering the Craving to Smoke: its Relation to Level of Presence and Nicotine Dependence | Ferrer-Garcia | Smoking | 2010 | Europe | Espagne | 1 | 8 women | 8 | 32.00 |  | 0 | 17 men (69%) | 17 | 68.00 | 25 | M=29,7, SD=13,4 | 29.7 | 13.4 | Adults | Former Smokers | Former Smokers | HMD | 5DT HMD 800 Series | a mouse | unspecified |  | Passive | testing iVR scenarios/ scene for smoking cessation intervention |
| Validation of VR-based Software for Binge Eating Treatment: Preliminary Data | Ferrer-Garcia | Binge eating | 2014 | Europe | Espagne | 1 | 30 women | 30 | 78.95 |  | 0 | 8 men | 8 | 21.05 | 38 | Unspecified |  |  | Adolescents |  | undergraduate student | HMD | Oculus Rift+ active navigation mode | | 20s*40 soit environ 13min total | 10 to 15 | Passive | VR for binge eating treamtment |
| Smoking a virtual cigarette increases craving among smokers | Garcia-Rodriguez | Smoking | 2013 | Europe | Espagne | 1 | 26 women | 26 | 57.78 |  | 0 | 19 men | 19 | 42.22 | 45 | M= 25.7; S= 8.9 | 25.7 | 8.9 | Adults | Smokers | Smokers | HMD | 5DT HMD 800 SERIES | a mouse | 6min | 5 to 10 | Active | studying smoking cravings when smoking in iVR vs just being exposed to environment |
| Efficacy of the Virtual Reality Intervention VR FestLab on Alcohol Refusal Self-Efficacy: A Cluster-Randomized Controlled Trial | Guldager | Alcohol | 2022 | Europe | Danemark | 1 | 192 women | 192 | 50.79 |  | 0 | 186 men | 186 | 49.21 | 378 | M= 15.67 | 15.67 |  | Adolescents |  | Highschool students (14 to 18 years old) | HMD | Oculus Quest | none | 15 min max | 10 to 15 | Passive | VR intervention for alcohol refusal self-efficacy in adolescents |
| Acceptability Evaluation of the Use of Virtual Reality Games in Smoking-Prevention Education for High School Students: Prospective Observational Study | Gui | Smoking | 2021 | Asia | Taiwan | 1 | 73 women | 73 | 56.15 |  | 0 | 57 men | 57 | 43.85 | 130 | M= 16.64; SD= 0.73 | 16.64 | 0.73 | Adolescents |  | Highschool students (under 17 mainly) | HMD | unspecified | 2 joysticks | 35-45 min "to complete educational tasks" | >30 | Active | Use of VR games to prevent smoke in regard to high school students |
| Moving Beyond Role-Play: Evaluating the Use of Virtual Reality to Teach Emotion Regulation for the Prevention of Adolescent Risk Behavior Within a Randomized Pilot Trial | Hadley | Risk behavior | 2019 | North America | US | 1 | 46 women | 46 | 54.12 |  | 0 | 39 men | 39 | 45.88 | 85 | M= 13; S= 0.85 | 13 | 0.85 | Adolescents |  | Middle school students (between 12-15 years old) | HMD | unspecified | unspecified | unspecified |  | Active | emotion regulation through VR roleplay sexual risk prevention in adolescents |
| Eliciting Affect via Immersive Virtual Reality: A Tool for Adolescent Risk Reduction | Hadley | Risk behavior | 2013 | North America | US | 1 | 19 women | 19 | 45.24 |  | 0 | 23 men | 23 | 54.76 | 42 | M= 14.54; SD= 1.13 | 14.54 | 1.13 | Adolescents |  | adolescents (between 12-16 years old) | HMD | eMagin z800 HMD system | none | 7 min | 5 to 10 | Passive | VR as a tool to initiate physiological arousal for risk perception in teens |
| A Virtual Reality Game to Change Sun Protection Behavior and Prevent Cancer:User-Centered Design Approach | Horsham | Sun protection | 2021 | Asia | Australia | 1 | 12 women | 12 | 66.67 |  | 0 | 6 men | 6 | 33.33 | 18 | 18-74 ans |  |  | Adults |  | adults | HMD | Oculus Quest | 2 controllers | 3 to 5 min | <5 | Active | Change sun protection behavior prevent cancer VR |
| Usability Study of a Novel Tool: The Virtual Cafeteria in Nutrition Education | Isgin-Atici | Nutrition | 2020 | Middle East | Turkey | 1 | 41 women (56%) | 41 | 56.16 |  | 0 | 32 men (44%) | 32 | 43.84 | 73 | M=22,2, SD=4,14 | 22.2 | 4.14 | Adults | Students | students | HMD | HTC Vive | 2 controllers | unlimited : some participant finished in 5min, some in 25 | unlimited 5 to 25 | Active | Virtual cafeteria pilot study for nutrition education for portion size |
| Development and Validation of a Virtual Reality-Based Training Program for Promoting Subjective Well-Being | Kim | well-being | 2020 | Asia | Korea | 1 | 0 | 0 | 0.00 |  | 0 | 36 men | 36 | 100.00 | 36 | M=23,7, SD=1,9 | 23.7 | 1.9 | Adults |  | adults | HMD | Oculus Rift | 1 controller | 40min | >30 | Active | Program to train subjective well-being |
| Using virtual reality to study food cravings | Ledoux | Nutrition | 2013 | North America | US | 1 | 55 women | 55 | 100.00 |  | 0 | 0 men | 0 | 0.00 | 55 | M=23,04, SD=3,75 | 23.04 | 3.75 | Adults | healthy BMI adults women | healthy BMI adults women | HMD | unspecified | 1 controller | 4*3 min donc 12min total avec 5 min breaks between VI modalities | <5 | Passive | Food craving study with VR |
| Application of Virtual Reality in an ED Primary Prevention Context : An Exploratory Study | Lemieux | Eating disorders | 2020 | North America | Canada | 1 | unspecified |  | 0.00 |  | 0 | unspecified |  | 0.00 | 37 | unspecified |  |  | Adolescents |  | adolescent between 11 to 14 years old) | unspecified | unspecified | unspecified | unspecified |  | Passive | primary prevention of ED in adolescent |
| The use of immersive stories to influence college students’ attitudes and intentions related to drinking and driving | Ma | Alcohol | 2020 | North America | US | 1 | 61 women | 61 | 57.01 |  | 0 | 46 men | 46 | 42.99 | 107 | M=19,06, SD= unspecified | 19.06 |  | Adults | Students | students | HMD | Samsung Gear VR + Samsung Galaxy S7 | none | 5min | 5 to 10 | Active | HMD vs iPad alcohol prevention on attitudes and intention to drive drunk |
| Modeling Dynamic Food Choice Processes to Understand Dietary Intervention Effects | Marcum | Nutrition | 2018 | Europe | UK | 1 | 221 women | 221 | 100.00 |  | 0 | 0 men | 0 | 0.00 | 221 | M=38, SD=5,62 | 38 | 5.62 | Adults | people suffering from obesity | mères en situation d'obésité ou de surpoids | HMD | unspecified | unspecified | unspecified |  | Active | Modeling food choice via VR buffet to understand dietary intervention |
| Effects of providing personalized feedback of child’s obesity risk on mothers’ food choices using a virtual reality buffet | McBride | Nutrition | 2013 | North America | US | 1 | 221 women | 221 | 100.00 |  | 0 | 0 men | 0 | 0.00 | 221 | M=38 | 38 |  | Adults | people suffering from obesity | mères en situation d'obésité ou de surpoids (mère d'enfants de 4-5ans) | HMD | unspecified | 1 controller | unspecified |  | Active | Child feeding behavior when aware of obesity risk |
| A self-administered virtual reality intervention increases COVID-19 vaccination intention | Mottelson | Vaccination | 2021 | Europe | Danemark | 1 | 20 women+ 7 non-binaire | 20 | 7.09 | 7 | 2.482269504 | 255 men | 255 | 90.43 | 282 | M= 28,9; SD= 9,7 | 28.9 | 9.7 | Adults | Unvaccinated adults | unvaccinated adults | HMD | Oculus Quest 1 or 2 | 2 controllers | 10 minutes | 10 to 15 | Active | VR intervention to enhance vaccination intention |
| Virtual reality for public health: a study on a VR intervention to enhance occupational injury prevention | Namkoong | Occupational injury | 2022 | North America | US | 1 | 130 women (44,7%) | 130 | 44.67 |  | 0 | 161 men (55,3%) | 161 | 55.33 | 291 | M=15,9, SD=1,4 | 15.9 | 1.4 | Adolescents |  | highschool student | HMD | unspecified | unspecified | 2,5 minutes | <5 | Passive | VR intervention upon tractor injury prevention |
| A Pilot Study to Develop a New Method of Assisting Children in Taking  Their Medication by Using Immersive Virtual Reality | Niki | Medication taking | 2021 | Asia | Japon | 1 | 7 women | 7 | 70.00 |  | 0 | 3 men | 3 | 30.00 | 10 | M= 21,8; SD= 0,8 | 21.8 | 0.8 | Adults |  | adults | HMD | Samsung Gear VR + Samsung Galaxy S7 | none | 4*30s environ 2mn | <5 | Passive | Assisting childrens taking their medication by using VR |
| Using immersive virtual reality to improve the beliefs and intentions of influenza vaccine avoidant 18-to-49-year-olds: Considerations, effects, and lessons learned | Nowak | Vaccination | 2020 | North America | US | 1 | 115 women | 115 | 67.25 |  | 0 | 56 men | 56 | 32.75 | 171 | 18-45ans ; (57,9% between 18 and 35 ans) |  |  | Adults | adults vaccine avoidant | adults vaccine avoidant | HMD | unspecified | 2 controllers | 5mn | 5 to 10 | Active | Using VR to improve the beliefs and intentions of influenza vaccine avoidant |
| Effects of Fruit and Vegetable Feeding Messages on Mothers and Fathers: Interactions Between Emotional State and Health Message Framing | Persky | Nutrition | 2018 | North America | US | 1 | 126 women | 126 | 66.32 |  | 0 | 64 men | 64 | 33.68 | 190 | M= 37,7; SD= 5,7 | 37.7 | 5.7 | Adults | Parents | parents of 4-5 years old children | HMD | unspecified | 1 controller | unspecified |  | Active | Health message framing and emotion induction on child feeding in iVR buffet |
| Investigating the Efficacy of Genetic, Environmental, and Multifactorial Risk Information When Communicating Obesity Risk to Parents of Young Children | Persky | Nutrition | 2021 | North America | US | 1 | 98 women | 98 | 51.58 |  | 0 | 92 men | 92 | 48.42 | 190 | M= 39,9; SD= 6,38 | 39.9 | 6.38 | Adults | Parents | parents of a 3 to 7 years old | unspecified | unspecified | unspecified | unspecified |  | Active | Family vs genetic risk to obesity on child feeding (VR buffet) |
| Can Physical Activity in Immersive Virtual Reality Be Attractive and Have Sufficient Intensity to Meet Health Recommendations for Obese Children? A Pilot Study | Polechonski | physical activity | 2020 | Europe | Poland | 1 | 4 women | 4 | 36.36 |  | 0 | 7 men | 7 | 63.64 | 11 | M=9,45; SD = 1,35 | 9.45 | 1.35 | Children | people suffering from obesity | children with obesity | HMD | HTC vive | 2 controllers | 15 minutes*2 with 30min break between | 15 to 20 | Active | Physical VR activity for obese children |
| A Virtual Reality-Based Self-Help Intervention for Dealing with the Psychological Distress Associated with the COVID-19 Lockdown: An Effectiveness Study with a Two-Week Follow-Up | Riva | well-being | 2021 | Europe | Italy | 1 | 25 women (62,5%) | 25 | 62.50 |  | 0 | 15 men (37,5%) | 15 | 37.50 | 40 | M= 30,28; SD= 11,69 | 30.28 | 11.69 | Adults | Adults having been in lockdown | Adults who experienced at least 2month of strict lockdown | HMD | basic low-cost cardboard VR headset (depending of what people have at home) | none | 10min/per day*7 days | 10 to 15 | Passive | VR self-help intervention to cope with psychological distress from COVID-19 |
| Enhancing Men's Awareness of Testicular Disorders Using a Virtual Reality Intervention: A Pre-Post Pilot Study | Saab | Testicular disorder | 2018 | Europe | Irland | 1 | 0 women | 0 | 0.00 |  | 0 | 49 men | 49 | 100.00 | 49 | 18-50ans; 21-30ans= 45% ; median age = 26 ans | |  | Adults |  | adults, with 56,7% of university students | HMD | unspecified | 1 controller | unspecified |  | Active | Rising mens' awareness of testicular disorder through VR |
| Simultaneous Exercise and Cognitive Training in Virtual Reality Phase 2 Pilot Study: Impact on Brain Health and Cognition in Older Adults | Sakhare | physical activity | 2021 | North America | US | 1 | 8 women | 8 | 66.67 |  | 0 | 4 men | 4 | 33.33 | 12 | M= 64,7; SD= 8,8 | 64.7 | 8.8 | Senior Adults |  | older adults | HMD | HTC vive pro | hands on the bike | 25-50 minutes | >30 | Active | Physical and cognitive training in VR, impact on elders' brain and cognition |
| Influence of Being Embodied in an Obese Virtual Body on Shopping Behavior and Products Perception in VR | Verhulst | Nutrition | 2018 | Asia | Japan | 1 | 2 women | 2 | 8.70 |  | 0 | 21 men | 21 | 91.30 | 23 | M=22,5; SD=0,79 | 22.5 | 0.79 | Adults | Students | students | HMD | Oculus CV1 | Optitrack suit | 20 min | 15 to 20 | Active | being embodied in an obese VR on food shopping behavior |
| A quasi-experimental test of a virtual reality game prototype for adolescent E-Cigarette prevention | Weser | Smoking (e-cigarette) | 2021 | North America | US | 1 | 9 women | 9 | 19.15 |  | 0 | 38 men | 38 | 80.85 | 47 | M= 14,23; SD= 0,914 | 14.23 | 0.914 | Adolescents |  | adolescents | HMD | Oculus Go VR | 1 controller | 1–2 times over the course of 1–2 weeks and accumulated an average of 93 min (SD = 26 min) of total play time. | >30 | Active | VR to prevent adolescent consumption of e-cigarettes |
| Evaluation of a virtual reality E-cigarette prevention game for adolescents | Weser | Smoking (e-cigarette) | 2021 | North America | US | 1 | 130 women + 2 "prefer not to say" | 130 | 46.59 | 2 | 0.716845878 | 146 men | 146 | 52.33 | 279 | M=12,45; SD= 0,57 | 12.45 | 0.57 | Adolescents |  | adolescents age 11 to 16 | HMD | Oculus Go VR | 1 controller | environ 20-40 minutes per session | >20 | Active | E-cigarette prevention game for adolescents |
| Acute Effects of Virtual Reality Exercise Biking on College Students’ Physical Responses | Zeng | physical activity | 2021 | North America | US | 1 | 34 women | 34 | 70.83 |  | 0 | 14 men | 14 | 29.17 | 48 | M= 23,58; SD= 3,39 | 23.58 | 3.39 | Adults | Students | students | HMD | Playstation VR | none | 20 minutes | >20 | Active | VR vs ebike vs traditional bike on RPE in college students |
| Acute Effect of Virtual Reality Exercise Bike Games on College Students' Physiological and Psychological Outcomes | Zeng | physical activity | 2017 | North America | US | 1 | 9 women | 9 | 75.00 |  | 0 | 3 men | 3 | 25.00 | 12 | M= 25,01; SD= 4,74 | 25.01 | 4.74 | Adults | Students | students | HMD | Playstation VR | none | 20 minutes | >20 | Active | Acute Effect of Virtual Reality Exercise Bike Games  on College Students’ |
| Using Virtual Reality to Improve the Quality of Life of Older Adults with Cognitive Impairments and their Family Members who Live at a Distance | Afifi | Quality of life and social interaction | 2023 | North America | US | 1 |  | 30 | 60 |  | 0 | 20 men | 20 | 40 | 50 | M = 83.10 , SD = 9.76 | 83.1 | 9.76 | Senior Adults |  | older adults | HMD |  |  | 15 minutes |  |  |  |
| The Influence of Billboard-Based Tobacco Prevention Posters on Memorization, Attitudes, and Craving: Immersive Virtual Reality Study | Bonneterre, Zerhouni, Boffo | Health promotion, tobacco prevention | 2024 | Europe | France | 1 |  | 100 | 82.6446281 |  | 0 | 20 men | 20 | 16.52892562 | 121 | M = 19.6 , SD = 3.7 | 19.6 | 3.7 | Adults | Students | students | HMD | Oculus Rift | 2 controllers |  |  |  |  |
| Effects of Immersive Virtual Therapy as a Method Supporting Recovery of Depressive Symptoms | Kiper et al. | Health promotion, recovery of depressive symptoms | 2022 | Europe | Poland | 1 |  | 30 | 50 |  | 0 | 30 men | 30 | 50 | 60 |  | 65.5 | 6.72 | Senior Adults |  | older adults | HMD |  |  |  |  | Active |  |
| Can the Virtual Reality Intervention VR FestLab Improve Alcohol Refusal Self-Efficacy? | Guldager, Kjær, Grittner, Stock | Health promotion, alcohol prevention | 2022 | Europe | Denmark | 1 |  | 186 | 49.20634921 |  | 0 | 192 men | 192 | 50.79365079 | 378 | M = 15,6 , SD = 2.56 | 15.6 | 2.56 | Adolescents |  | adolescents (between 15-18) | HMD | Oculus Quest | 2 controllers | 15 minutes |  | Active |  |
| Subjective Experience on Virtual Reality-Assisted Mental Health Promotion Program | Ko, Lim, Park, Kim et al. | Mental health promotion through VR | 2024 | Asia | South Korea | 1 |  | 72 | 75 |  | 0 | 24 men | 24 | 25 | 96 | M = 72.8, SD = 17.2 | 72.8 | 17.2 | Senior Adults |  | older adults | HMD |  |  | 5 minutes |  | Active | The VR-assisted mental health promotion program effectively reduced depressive and stressful feelings, particularly among the high-risk group. |
